# Banxia Xiexin decoction: A review on phytochemical, pharmacological, clinical and pharmacokinetic investigations

**DOI:** 10.1097/MD.0000000000034891

**Published:** 2023-09-01

**Authors:** Zehua Zhou, Rui An, Lisha You, Kun Liang, Xinhong Wang

**Affiliations:** a Shanghai University of Traditional Chinese Medicine, Shanghai, China.

**Keywords:** Banxia Xiexin decoction, clinical, parmacological, pharmacokinetic, phytochemical, traditional Chinese medicine

## Abstract

Banxia Xiexin decoction (BXD), a famous traditional Chinese prescription constituted by Pinelliae Rhizoma, Zingiberis Rhizoma, Scutellariae Radix, Coptidis Rhizoma, Ginseng Radix et Rhizoma, Jujubae Fructus and Glycyrrhizae Radix et Rhizoma Praeparata Cum Mell, has notable characteristics of acrid-opening, bitter down-bearing and sweet-tonification, interfering with tumors, gastrointestinal diseases, central nervous system diseases and much more. Based on the wide clinical applications, current investigations of BXD focused on several aspects: chemical analysis to explore the underlying substrates responsible for the therapeutic effects; basic studies on pharmacological actions of the whole prescription or of those representative ingredients to demonstrate the intriguing molecular targets for specific pathological processes; pharmacokinetic feature studies of single or all components of BXD to reveal the chemical basis and synergistic actions contributing to the pharmacological and clinically therapeutic effects. In this review, we summarized the main achievements of phytochemical, pharmacological, clinical and pharmacokinetic profiles of BXD and its herbal or pharmacologically active chemicals, as well as discussions of our understanding which further reveals the significance of BXD clinically.

## 1. Introduction

Herbal formula, being the most popular therapeutic approach of traditional Chinese medicine (TCM), has been recorded in ancient medical literature with fixed herbal components, definite curative effects, and acceptable adverse effects.^[1]^ Banxia Xiexin decoction (BXD) (Hange-shashin-to in Kampo medicine and Banha-sasim-tang in Korean), a well-known classic TCM formula, was first described in Zhongjing Zhang’s treatise “*Shang Han Lun*” in the East Han dynasty (219 A.D.). The clinical application of BXD has gradually expanded from the symptoms of TCM to diseases of western medicine, and its use has also spread to other countries. At present, BXD has been widely used in the clinical practices to treat gastroenteritis, inflammatory bowel diseases, diarrhea, etc.^[2–4]^ In China, thin-layer chromatography and microscopy have been employed to establish the quality standard of BXD for decades. The contents of flavonoids, alkaloids and saponins in BXD have been determined.^[5]^ In other Asian countries, BXD was approved to treat functional dyspepsia or ulcerative diseases in the gastrointestinal tract by Ministry of Health, Labour and Welfare of Japan and Korean Food and Drug Administration.^[6,7]^ In this review, we summarized the main achievements of phytochemical, pharmacological, clinical and pharmacokinetic profiles of BXD and its herbal or pharmacologically active chemicals. We also discussed our understanding of BXD to further reveals the clinical significance of BXD.

## 2. Composition and general pharmacological action of BXD

In TCM theory, the composition of BXD represents the harmony between acrid-opening, bitter down-bearing and sweet-tonification features. BXD shows the ability to harmonize liver and spleen, calm cold and heat, eliminate mass and resolve hard mass. This ability is achieved by 7 common crude herbs, Pinelliae Rhizoma (BX), Zingiberis Rhizoma (GJ), Scutellariae Radix (HQ), Coptidis Rhizoma (HL), Ginseng Radix et Rhizoma (RS), Jujubae Fructus (DZ) and Glycyrrhizae Radix et Rhizoma Praeparata Cum Mell (ZGC) (Table 1).^[7]^ Following the strict principle of “sovereign, minister, assistant and courier,”^[8]^ which was derived from “Huangdi’s Internal Classic” to improve the efficacy of TCM and to reduce toxics or adverse effects by combining different types of herbs. In BXD, HL serves as “sovereign” to treat the main disease. HQ acts as “minister” to enhance the effects of “sovereign.” BX and GJ perform the role of “assistant” to reduce possible toxicity of “sovereign” or “minister” and treat accompanying symptoms. RS, ZGC, and DZ are “courier” to guide the other herbs to the target organs.

**Table 1:** The composition of BXD.

Species	Chinese name	Plant part	Grams, g
Pinelliae Rhizoma	Ban xia	Tuber	12
Scutellariae Radix	Huang qin	Root	9
Zingiberis Rhizoma	Gan jiang	Rhizome	9
Ginseng Radix et Rhizoma	Ren shen	Root and rhizome	9
Glycyrrhizae Radix et Rhizoma Praeparata Cum Mell	Zhi gan cao	Root and rhizome	9
Coptidis Rhizoma	Huang lian	Rhizome	3
Jujubae Fructus	Da zao	Fruit	16
Total amount			67

BXD = Banxia Xiexin decoction.

In terms of material basis, the main components in each herb are the functional materials exhibiting physiological and biochemical activities. Briefly, gingerol and shogaol in GJ are the main active components with anti-inflammatory, antioxidant, and anticancer activities. They can also modulate the hypothalamo-pituitary-adrenal (HPA) axis and central nervous system (CNS).^[9]^ The abundant flavonoids in HQ have a wide range of biological activities, such as anti-inflammation, antibacterial effect, lowering of blood lipid and cholesterol, and prevention and treatment of hypertension. Plentiful alkaloids in HL have multiple medicinal effects including antiviral, antibacterial, anti-inflammatory, anti-cardiovascular, anti-oxidative, and anti-diarrhea effects. The combination of HL and HQ showed great potential to further develop into a promising clinical treatment approach for *Helicobacter pylori* (Hp) infection.^[10–12]^ The main active ingredient in RS is ginsenoside that has anti-tumor function, improves learning and memory, and has activities for anti-aging, anti-oxidation, and scavenging of free radicals in the body.^[13,14]^ Glycyrrhizic acid and liquiritin in ZGC possesses anti-inflammatory, anticancer, and anti-ulcer activities.^[15,16]^ Furthermore, the beneficial effects of these active ingredients from different herbs might have coordinated and harmonized to reach overall responses that enable BXD to hit multiple targets simultaneously and exert synergistic therapeutic effects.^[17]^ Nevertheless, owing to a lack of TCM theories such as the theoretical mechanisms of diseases, it is hard to decompose recipes and reveal the complex interactions of pharmacodynamic substance groups in BXD.

Due to the increase of clinical applications, more and more researchers begin to explore the underlying pharmacological actions and mechanisms of BXD via advanced biotechnology. Recent studies suggest that BXD possesses therapeutic effects in different pathological aspects. Additionally, with the development of technology, more and more chemical components of BXD have been identified. To guide the studies further, it is necessary to summarize these past findings of BXD. In this review, we summarized the phytochemical, pharmacological, clinical, and pharmacokinetic investigations in recent years.

## 3. Phytochemical investigation of BXD

Given that the components of TCM formulas are complex and not all of them have pharmacological effects, separating and identifying such pharmacodynamic components is crucial. Recent studies have shown that the three main active components of BXD are alkaloids, flavonoids, and saponins. As a result, these three are recognized as markers for the quality control of BXD.^[5,18]^ With the rapid development of modern analysis technology, many researchers have actively explored the chemical components in BXD and established qualitative and quantitative detection methods for some of its active components. 18 major peaks (jatrorrhizine, palmatine, berberine, baicalin, wogonoside, baicalein, wogonin, coptisine, oroxin A, scutellarin, liquiritin, liquiritigenin, isoliquiritin, isoliquiritigenin, Ginsenoside Rb_1_, Ginsenoside Rg_1_, Ginsenoside Re and glycyrrhizic acid) in the chromatogram of BXD extracted with water were identified by using UPLC-MS/MS. Among them, baicalin, wogonoside, berberine, coptisine and liquiritin were determined as the main active compounds in BXD.^[5]^ Moreover, TCM are originally administered in the form of decoctions prepared by boiling crude drugs according to formulas in classic literature. In comparison with the methanol-diluted decoction or extract granule (produced by concentrating and drying the decoction, which is usually made in industrial scale) of BXD by IP-HPLC, the result verified that the extract granule showed a low content of marker components in BXD.^[18]^ Here, the chemical compounds of BXD were summarized and categorized for further separation and analysis.

## 4. Alkaloids

**Figure 1.**
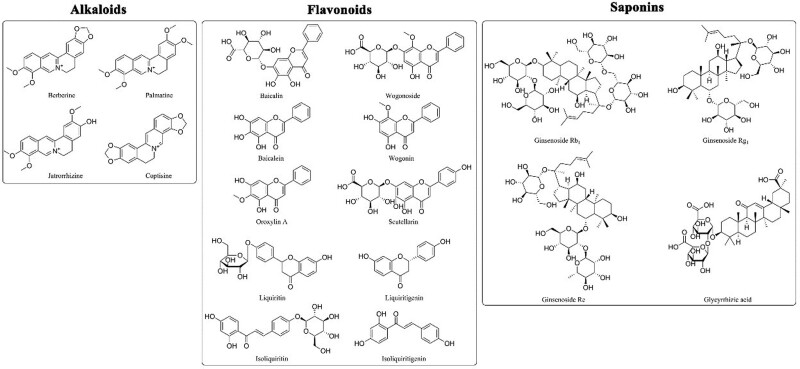
Structures of the main constituents in BXD. BXD = Banxia Xiexin decoction.

Alkaloids are nitrogen-containing organic components, which give BXD its bitter taste. The main alkaloids in BXD come from HL. Among them, berberine, palmatine, coptisine and jatrorrhizine (Fig. 1) are regarded as the major bioactive components of HL in BXD.^[5,19–27]^ Moreover, the quantitative determination of berberine, palmatine and coptisine is a very important index to evaluate the quality of HL.

Due to the structural characteristics, alkaloids tend to have poor absorptions. For example, berberine, as a quaternary ammonium alkaloid with conjugated double bonds, usually has strong rigidity and poor solubility. Moreover, berberine is the substrate of P-glycoprotein, which is an efflux transporter.^[28]^ Additionally, after intragastric administration, most berberine was excluded by the gastrointestinal tract and metabolized in various pathways.^[29]^ Therefore, it is hard for alkaloids to accumulate in the body even with a long-term administration.

## 5. Flavonoids

So far, more than 40 flavonoids have been found from HQ in the form of aglycones and glycosides, making flavonoids the most bioactive compounds in HQ.^[30]^ The characteristic chemical components of HQ in BXD include baicalin, baicalein, wogonoside, wogonin, oroxylin A and scutellarin (Fig. 1).^[31]^ By UPLC-MS/MS analysis, the contents of the above 6 flavonoids in BXD was 11.9 mg/g, 0.252 mg/g, 2.04 mg/g, 0.166 mg/g, 0.0458 mg/g, and 0.224 mg/g respectively.^[5]^ Relatively speaking, flavonoids were more easily absorbed compared to alkaloids. Due to the ease to combine with glucuronic acid or sulfuric acid to form two-phase metabolism, the plasma concentration-time curves of flavonoids clearly demonstrated bimodal features.^[32]^

Besides, 4 flavonoids of ZGC in BXD, namely liquiritin, liquiritigenin, isoliquiritin and isoliquiritigenin (Fig. 1), are the other main components that can be observed.^[5]^ Many studies have shown that these flavonoids are one of the most important active substances in ZGC, which play important roles in anti-ulcer, antispasmodic and other physiological functions of ZGC.^[33]^ However, the contents of the above 4 flavonoids in BXD was low, about 0.349 mg/g, 0.0333 mg/g, 0.0787 mg/g, and 0.0161 mg/g, respectively.^[5]^

## 6. Saponins

Another major compounds detected in BXD are saponins, most of which are from RS (Ginsenoside Rb_1_, Ginsenoside Rg_1_, and Ginsenoside Re) (Fig. 1).^[5]^ RS contains a variety of bioactive monomers such as mineral oils, ginseng saponins, fatty acids, and polysaccharides.^[34]^ Among them, the triterpene saponins, also known as ginsenosides, are mainly responsible for the pharmacological effects of RS. Ginseng saponins are usually hard to absorb by the body through the intestines due to their hydrophilicity. Only small amount can be absorbed in the gastrointestinal tract following oral intake. According to reports, saponins can be degraded into secondary glycosides or aglycones by the intestinal flora through hydrolysis of the glycosidic bonds and modulate their pharmacokinetics after orally administration, which further impacts their pharmacological efficacies in turn.^[35]^

Additionally, BXD contains a kind of triterpenoid saponin,^[5]^ namely glycyrrhizic acid (Fig. 1), which is isolated from the roots and rhizomes of ZGC. Glycyrrhizic acid is the major characteristic constituents of ZGC.^[36]^ A mixture of potassium and calcium salts of glycyrrhizic acid, glycyrrhizin, is 50 times as sweet as sugar and often used as sweetener or flavor enhancer.^[33]^

## 7. Other chemical components

Other substances abundant in HL are lignans, polyphenolic substances derived from phenylalanine by dimerizing substituted cinnamic alcohols, which have many structures such as benzofurans, furofurans, tetrahydrofurans and arylnaphthanlenes.^[37–40]^ Besides, phenylpropanoids,^[37,39]^ phenolic compounds, saccharides, and steroids have been isolated from HL.^[39,41–44]^ Diterpenoids^[45,46]^ and essential oils^[47]^ were reportedly discovered in HQ. 10-gingediol, 6-gingerol, jaranol and α-curcumene were found in GJ. Moreover, fatty acids such as zizyberenalic acid and zizyberanal acid, mainly from DZ, phenolic acids (L-pipecolic acid and adenosine) were detected as well.^[48]^

## 8. Pharmacological effects

**Figure 2.**
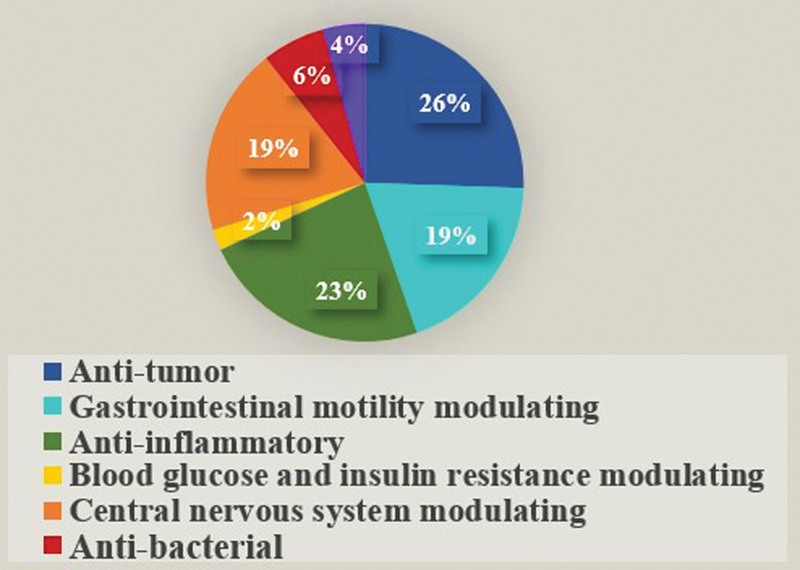
Proportions of the pharmacological effects of BXD. BXD = Banxia Xiexin decoction.

Thanks to the progress of modern pharmacology and biological technologies, increasing evidence has shown the pleiotropic therapeutic effects of BXD on gastrointestinal diseases, cancer, CNS diseases, etc (Fig. 2; Table 2).

**Table 2:** Diseases and regulatory mechanisms of BXD in research.

Diseases	Type of research	Patients or model	Mechanisms	Refs.
Functional dyspepsia	Clinical	Patients with functional dyspepsia	/	^[7]^
	Basic	Loperamide induced functional dyspepsia mice	Modulating peristalsis via activation of ICCs and the smooth muscle cells in the stomach	^[49]^
Gastric cancer	Basic	SNU-16 cells	Inhibiting Wnt/β-catenin signaling pathway, thus inhibiting SNU-16 cell activity and clone formation, promoting oxidative stress, and inducing apoptosis	^[50]^
	Basic	Clinical Specimens Gastric cancer bearing nude mice AGS cells	Inhibiting the proliferation and promoting the apoptosis of gastric cancer cells by indirectly regulating the expression of PD-L1 through multiple pathways and targets	^[4]^
	Systematic review and meta-analysis	/	/	^[51]^
	Basic	GC9811- P cells	Inhibiting the proliferation, invasion and metastasis of GC9811-P cells, which might be associated with blocking peritoneal metastasis of gastric cancer	^[52]^
	Basic	BGC-823 cells	Inhibiting proliferation of BGC-823 cells and inducing cell apoptosis (especially the combination of pungent-opening or bitter-descending components in BXD)	^[53]^
	Basic	Clinical Specimens Gastric cancer bearing Nude mice AGS cells	Influencing the drug sensitivity of gastric cancer cells by regulating the expression of MGMT via IL-6/JAK/STAT3-mediated PD-L1 activity	^[54]^
Ulcerative colitis	Network pharmacology analysis	TNBS-induced ulcerative colitis rats	/	^[55]^
	Basic	DSS-induced chronic ulcerative colitis mice	Inhibiting the activation of NF-κBp65 and increasing the expression of Nrf2 in colorectums of mice	^[2]^
	Basic	Oxazolone-induced colitis mice	Decreasing the mRNA expression of IL-5 and IL-13	^[56]^
	Network pharmacology analysis	/	/	^[57]^
Pouchitis	Clinical	Patients with chronic pouchitis	/	^[58]^
Gastritis and gastric precancerous lesion	Network pharmacology analysis	Ethanol-induced chronic gastritis rats	/	^[59]^
	Systematic review and meta-analysis	/	/	^[60]^
	Systematic review and meta-analysis	/	/	^[61]^
	Basic	Chronic atrophic gastritis rats	Targeting the Notch signaling pathway	^[62]^
	Systematic review and meta-analysis	/	/	^[63]^
	Clinical	Patients with chronic gastritis		^[64]^
Postprandial distress syndrome	Clinical	Patients with Wei-Pi syndrome	/	^[65]^
Colon cancer	Clinical	Patients with stage III colon cancer	/	^[66]^
	Basic	Colon cancer bearing nude mice	Regulating oxidative stress and inflammation, targeting apoptosis and increasing the levels of MAPK/NF-κB pathway	^[67]^
	Basic	Xenograft nude mouse model with HCT116 cells	Inhibiting the expression of IL6 and TNFα proinflammatory factors and enhance the expression of CASP3 apoptotic protein	^[68]^
Hp-associated diseases	Systematic review and meta-analysis	/	/	^[69]^
	Basic	Hp-treated GES-1 cells	Regulating the TGF-β/Smad signaling pathway by inhibiting the expression of TGF-β1 and Smad3, and increasing the expression of Smad7	^[70]^
	Basic	Hp bacteria collected and isolated from patients’ gastric mucosa	Bacteriostatic effect on the bacteria resistance of Hp strains	^[71]^
	Systematic review and meta-analysis	/	/	^[72]^
CPT-11-induced intestinal toxicity	Basic	CPT-11-induced diarrhea mice	Establishing the components in BXD that were bioactive and capable of preventing CPT-11-induced diarrhea	^[73]^
	Clinical	Patients with recurrent small cell lung cancer undergoing chemotherapy regimens including CPT-11	/	^[3]^
Diabetic gastroparesis	Network pharmacology analysis	/	/	^[74]^
	Basic	STZ induced diabetic gastroparesis model rats	Modulating PLC-IP3-Ca2+/NO-cGMP-PKG pathway by increasing in expressions of PLC, IP3, NO, nNOS, cGMP and PKG and promoting contraction of gastric smooth muscle cell, as well as an increase in [Ca^2+^]_i_	^[75]^
	Basic	STZ induced diabetic gastroparesis model rats	Promoting the expression of positive ICCs and SCF	^[76]^
	Systematic review and meta-analysis	/	/	^[77]^
Advanced hepatocellular carcinoma	Clinical	Patients with advanced hepatocellular carcinoma	/	^[78]^
Depression	Network pharmacology analysis	/	/	^[57]^
Type 2 diabetes mellitus	Basic	Min6 cells	Inhititing apoptosis and improving insulin secretory function through modulation of the PI3K/AKT pathway and the downstream FOXO1	^[79]^
Gallbladder cancer	Clinical	Patient with advanced gallbladder cancer	/	^[80]^
Irritable bowel syndrome	Network pharmacology analysis	/	/	^[81]^
Alzheimer’s disease	Basic	APPswe/PS1dE9 mice	Improving insulin signaling, glucose metabolism and synaptic plasticity	^[82]^
Gastroesophageal reflux disease	Systematic review and meta-analysis	/	/	^[83]^
	Systematic review and meta-analysis	/	/	^[84]^
	Basic	Reflux esophagitis rats	Repair effects on damaged mucosa, increasing the pressure of esophageal sphincter, and inhibiting gastric acid	^[85]^
	Basic	Reflux esophagitis rats	Regulating the synthesis and secretion of NT	^[86]^
	Clinical	Patients with laryngopahryngitis caused by gastroesophageal reflux	/	^[87]^
	Systematic review and meta-analysis	/	/	^[88]^
Lung cancer	Basic	A549 cells	Inducing apoptosis by mechanisms involving an increase in Bax, a decrease in Bcl-2, activation of procaspase-9 and -3, and PARP cleavage	^[89]^
Gastric ulcer	Basic	Acetic acid-induced gastric ulcer rats	Upregulating Leptin and inhibiting ET-1	^[90]^
	Basic	Water immersion-restraint stress induced gastric ulcer rats	Increasing the expression of somatostatin	^[91]^
	Basic	Water immersion-restraint stress induced gastric ulcer rats	Increasing the content of gastric mucin	^[92]^
Gastrointestinal motor dysfunction	Basic	Circular smooth muscle of the rat distal colon	Inhibiting both spontaneous contractions and cholinergic responses by blocking L-type Ca^2+^ channels	^[93]^
Diarrhea	Baisc	5-Fluorouracil-induced diarrhea mice	Active ingredients of BXD, baicalein and 6-gingerol, prevent the development of neutrophil recruitment and diarrhea by the inhibition of NF-κB activity	^[94]^
	Basic	Castor oil induced diarrhea mice	*Scutellariae Radix, Glycyrrhizae Radix, Ginseng Radix* and *Coptidis Rhizoma* derived components contribute to the efficacy of inhibiting the expression of COX-2	^[95]^
Postoperative sore throat	Clinical	Postoperative sore throat in patients undergoing laparoscopic surgery	/	^[96]^
Stress-related diseases	Basic	Volunteers under venipuncture stress	Suppressing plasma levels of Neuropeptide Y under venipuncture stress	^[97]^
	Basic	Volunteers under venipuncture stress	Suppressing plasma adrenocorticotropic hormone and cortisol levels under stress	^[98]^
Spleen-deficiency constipation	Basic	Senna water decoction induced spleen-deficiency constipation mice	Regulating the intestinal microorganisms and enzyme activities	^[99]^
Polycystic ovarian syndrome with insulin resistance	Basic	Letrozole induced polycystic ovarian syndrome with insulin resistance rats	Adjusting the disorder of intestinal microbiota and then improving the metabolic disorder. *Akkermansia* may play an important role in the treatment.	^[100]^

BXD = Banxia Xiexin decoction, DSS = dextran sulfate sodium salt, Hp = Helicobacter pylori, ICCs = interstitial cells of Cajal, MGMT = 6-O-methylguanine-DNA methyltransferase, PD-L1 = programmed cell death-ligand 1, SCF = stem cell factor, STZ = streptozotocin, TNBS = 2,4,6-trinitrobenzenesulfonic acid.

## 9. Anti-tumor

According to Zhongjing Zhang, some cancer-like symptoms like “Yong, Yang, Chuang and Zhong” are usually induced by retention of multiple pathogens including heat and dampness. Induction of cell cycle progression, avoidance of apoptosis, and activation of the cell survival pathway are three key factors necessary for tumor growth.^[101]^ Modern studies indicated that BXD could inhibit the tumor growth and suppress proliferation of cancer cells by interfering with these processes.

One of the main mechanisms of colon cancer is the apoptosis and necrosis of intestinal epithelial cells induced by inflammatory factors. In a model of colon cancer bearing nude mice, BXD was shown to decrease SOD and increase MDA and pro-inflammatory cytokines. Moreover, western blotting revealed that the protein expressions of Bax, Caspase-3, Caspase-9 were increased by BXD, while Bcl-2 was decreased. BXD also decreased Nrf-2 and HO-1, and increased the levels of MAPK/NF-κB pathway in tumor tissue.^[67]^ Ma et al explore the molecular mechanism of BXD in treating colon cancer utilizing network pharmacology. They identified a total of 55 bioactive components and 136 cross-targets of BXD. According to the enrichment analysis, the oxidate stress and diet were the critical factors of colon cancer occurrence, and AGE-RAGE signaling pathway plays a key role. The study uncovered that BXD prevented colon cancer from progressing malignantly, which may be due to various compounds (berberine, quercetin, baicalein, etc.), multiple targets (Bcl-2, Bax, IL-6, TNF-α, CASP3, etc.), and multiple pathways (human cytomegalovirus infection, AGE-RAGE signaling pathway in diabetic complications, etc.). They also proved that BXD have the ability to inhibit tumor growth and induce tumor cell apoptosis in the HCT116 cell-xenograft nude mice model.^[68]^

Previous studies have revealed that the expression of programmed cell death-ligand 1 (PD-L1) is increased in gastric cancer (GC), suggesting that PD-L1 might play a critical role in the progress of GC.^[102]^ According to a present study on the regulatory mechanism of BXD acting on PD-L1 in GC, BXD could inhibit the expression of PD-L1 through multi-target and multi-pathway, which would affect the proliferation and apoptosis of cancer cells.^[4]^ Furthermore, the mechanism of drug sensitivity of BXD to GC was also investigated in vitro and in vivo. The results indicated that BXD influenced the drug sensitivity of GC cells by regulating the expression of 6-O-methylguanine-DNA methyltransferase. This process was executed via the non-PD-1 targeting of PD-L1, which is mediate by IL-6/JAK/STAT3 signaling.^[54]^ Additionally, by culturing SNU-16 cells with different concentrations of BXD serum (25, 50, 100 μL/mL), researchers found that, with increasing BXD concentration, the clonogenic ability of cells was inhibited. Moreover, BXD could also inhibit the activation of Wnt/β-catenin signaling pathway, which was partially inhibited by a Wnt/β-catenin signaling pathway activator.^[50]^ Additionally, some reports also confirmed the anti-tumor effect of BXD in other GC cells, such as GC9811-P cells and BGC-823 cells. The mechanism may be associated with inhibiting the proliferation, invasion and metastasis of GC cells, and inducing cells apoptosis.^[52,53]^

A study examined the synergistic effects of BXD extracts and cisplatin (a common drug for the treatment of lung cancer) using A549 lung cancer cell line.^[89]^ BXD was discovered to suppress the proliferation of A549 cells. Moreover, treatment with either BXD or cisplatin, or both promoted the apoptosis of cells, while pretreatment with Z-VAD-FMK (a nonspecific caspase inhibitor) inhibited these effects. These findings suggest that BXD may have the ability to sensitize the apoptosis of A549 cells induced by cisplatin, likely via increasing the expression of Bax, decreasing Bcl-2, activating procaspase-9 and -3, and PARP cleavage.

## 10. Gastrointestinal motility

Recorded in “*Shang Han Lun*,” BXD is also a well-known formula to treat syndrome of feeling fullness and distension in the upper abdomen. There was plenty of evidence demonstrating BXD could mitigate gastrointestinal motility disorders.

Patients with diabetes mellitus may also experience diabetic gastroparesis (DGP), a sort of chronic gastrointestinal motility impairment. The common symptoms include early satiety, nausea, abdominal distension, vomiting and loss of appetite. A study employed network pharmacology to identify the common and intersection targets of BXD and DGP, analyzed the pathways, and ultimately explored the action principle of BXD on DGP. According to the findings, 30 possible targets of BXD against DGP were screened out of 730 differentially expressed genes of DGP. 60 compounds in BXD, including the key active ingredients such as quercetin, wogonin, baicalein, beta-sitosterol, and kaempferol, have been shown to have a therapeutic impact on DGP. 68 pathways including TNF signaling pathway, IL-17 signaling pathway, and AGE-RAGE signaling pathway were significantly enriched, which provides a reference for further studies.^[74]^ According to Wang et al, treatment with high- and medium-dose BXD (4.6 and 9.2 g/kg/d) greatly alleviated DGP induced by streptozotocin (STZ) in rats. The mechanism of BXD might be related to the increases in expressions of PLC, IP3, NO, nNOS, cGMP and PKG, stimulation of contraction of gastric smooth muscle cells, and an increase in [Ca^2+^]_i_.^[75]^ Furthermore, recent studies have shown that interstitial cells of Cajal (ICCs) are gastrointestinal pacing cells, and the changes in their structure and number may be an important cause of gastrointestinal motility disorders.^[103]^ As a specific marker of ICCs, stem cell factor (SCF) plays a crucial role in the growth, development, and phenotype maintenance of ICCs. Jiang et al found that BXD could promote the expression of positive ICCs and SCF. It could also improve the gastric motility by partially inversing abnormal changes of gastric antral ICCs and SCF.^[76]^

A significant portion of the world’s population is affected by gastroesophageal reflux disease (GERD), which is characterized as a gastroesophageal motility disorder that manifests when the reflux of stomach contents results in bothersome gastroesophageal symptoms and/ or complications. Evidence from a systematic review and meta-analysis has demonstrated the validity of BXD in the treatment of GERD.^[84]^ In another study, rat models of GERD were treated with BXD following modified partial cardia muscle incision and pylorus ligation of external parts. The therapeutic impact became increasingly clear as the therapeutic course progressed. The therapeutic effect of BXD might be accomplished by repairing injured mucosa, inhibiting the secretion of gastric acid, and increasing the pressure of esophageal sphincter.^[85]^

Moreover, a study evaluated the pharmacological effects and the underlying mechanisms of BXD on the functional dyspepsia (FD) in loperamide-induced mice. The results showed that the loperamide injection significantly delayed stomach emptying, while pretreatment with BXD substantially alleviated this peristaltic dysfunction. Furthermore, pretreatment with BXD significantly attenuated the loperamide-induced inhibition of the expression of c-kit, nNOS, and contraction-related gene including the 5HT_4_ receptor, anoctamin-1, ryanodine receptor 3 and smooth muscle myosin light chain kinase. BXD also significantly alleviated the alterations in gastrointestinal motility.^[49]^

Widely used as an anti-cancer medication, 5-fluorouracil (5-FU) is also known to induce diarrhea. Researchers investigated the effects of BXD and its active ingredients on the upregulation of CXCL1 induced by 5-FU in colon tissue, and their effects on 5-FU-induced diarrhea mice. The findings demonstrated that BXD, particularly baicalein and 6-gingerol, decreased the mRNA expression of CXCL1 via activating NF-κB in vitro, and attenuated the development of neutrophil recruitment and 5-FU-induced diarrhea in vivo.^[94]^

## 11. Anti-inflammatory

According to TCM theory, heat and toxins, both endogenous and exogenous, are pathogenic factors of inflammation. In some ways, inflammatory factors released by inflammations are viewed as toxins leading to the heat syndromes appearing in conjunction with inflammatory and allergy reactions. It is conceivable that BXD might exhibit its powerful anti-inflammatory ability via inhibiting inflammatory cytokines and prostaglandin E_2_.^[104,105]^ Numerous studies have demonstrated that BXD is effective in treating various inflammatory diseases, especially gastritis and inflammatory bowel disease.

Chronic atrophic gastritis (CAG) is a progressive digestive disease characterized by the loss of normal glandular epithelium in the stomach lining as a result of ulceration, erosion and chronic inflammation, leading to mucosal injury and dysfunction.^[106]^ In a rat model of CAG, BXD at all three doses (6, 12, and 24 g/kg/d) was found to significantly reduce inflammatory cell infiltration, improve gastric mucosal morphology, augment thickness of the gastric mucosa, increase number of gastric glands, enhance mucosal expression of proliferating cell nuclear antigen, and elevate serum gastrin levels in CAG rats. The anti-inflammatory effect of BXD may be due to the downregulation of Notch1/2 signaling pathway.^[62]^ Ji et al demonstrated the effectiveness of BXD in the treatment of chronic gastritis by integrating network pharmacology with Ultra Performance Liquid Chromatograph-linear trap quadrupole-Orbitrap mass spectrometry (UPLC-LTQ-Orbitrap MS). Overall, BXD reduced the levels of TNF-α, IL-2, IL-8, and LDH while alleviating chronic gastritis in rats. 14 active compounds in BXD and 38 potential targets were screened out. According to KEGG pathway and PPI network analysis, several signaling pathways including NF-κB, HIF-1, PI3K-Akt, and JAK-STAT may play a role in the effects of BXD treating for chronic gastritis. By using surface plasmon resonance and molecular docking techniques, it was confirmed that multiple important targets, including ICAM-1, PPAR-γ and MAPK14, were related to crucial BXD compounds.^[59]^

Ulcerative colitis (UC) is a kind of inflammatory bowel disease, which is intimately associated with inflammation.^[107]^ A study showed that treatment with BXD could significantly attenuated dextran sulfate sodium salt-induced experimental colitis in mice. The mechanism of BXD might involve inhibiting the activation of NF-κB p65 and increasing the expression of Nrf2 in colorectums of mice.^[2]^ In a model of BALB/c mice with oxazolone-induced UC, the disease activity index (DAI) and histopathological inflammation score were dramatically reduced following intragastric administration of BXD extracted with water. Additionally, the mRNA expression levels of IL-5 and IL-13 in the colonic tissue showed significant decrease.^[56]^ Wang et al studied the pharmacological effects of BXD by using UC rats induced by 2,4,6-trinitrobenzenesulfonic acid. The extracts of BXD were prepared following either traditional or modern method. Based on the network pharmacology analysis, some key active ingredients (quercetin, baicalein, wogonin, and baicalin) related to the anti-UC/inflammation were identified, and the interactions between their candidate targets and UC targets were investigated. The results indicated that both traditional and modern extraction method might alleviate the severity of UC to varying extents.^[55]^ By using a systems pharmacology strategy, a study investigated active principles, drug targets and key pathways to reveal multicomponent synergy mechanism of BXD in treating irritable bowel syndrome (IBS), another kind of inflammatory bowel disease. As a result, 35 candidate components and 16 related key targets were screened out. 27 crucial pathways could modulate the biological processes, closely associated to pathological mechanism of IBS, including the synthesis of inflammatory mediators, smooth muscle relaxation and synaptic plasticity. The crosstalk interactions were uncovered between TNF signaling pathway, Dopaminergic synapse and cGMP-PKG signaling pathway, which might have therapeutic effects on IBS. There were 3 main therapeutic modules participated in the synergy molecular mechanisms of BXD, namely the inhibition of inflammatory reaction, the regulation of intestinal function and the improvement of psychological regulation.^[81]^

## 12. Blood glucose and insulin resistance modulating

In TCM theory, the symptom of wasting-thirst refers to syndrome *X*, which is induced by excessive “heat” dissipating bodily fluids. Recently, it was believed that the internal heat is the main pathogenic factor for the occurrence of type 2 diabetes mellitus (T2DM). Additionally, insulin resistance or deficiency in T2DM leads to the elevation of fasting and postprandial glucose and lipid levels, resulting in a series of vascular complications finally.

In the progress of T2DM, pancreatic β-cell plays an important role.^[108]^ Increased pancreatic β-cell apoptosis results in the loss of β-cell in T2DM, which ends in progressive deterioration and finally failure of β-cell function. Du et al found that preincubation with BXD could significantly improve *tert*-butyl hydroperoxide (t-BHP)-induced proliferation inhibition and apoptosis and enhance glucose-stimulated insulin secretion. Pretreatment with BXD countered the increase of reactive oxygen species and the inhibition of antioxidant enzymes induced by t-BHP. In t-BHP-induced MIN6 cells, BXD was found to promote the phosphorylation of AKT and FOXO1. Furthermore, BXD tempered the expression of apoptosis related indicators Bax, P27, and Caspase-3, which could be reversed by PI3K inhibitor LY294002.^[79]^

## 13. CNS modulating

It is believed that the disorder of CNS is closely related to various diseases, even disorder of digestive system. The classic clinical symptoms include dysfunction of learning, depression and memory. In synaptic transmission, neurotransmitters, specific chemical substances, act as the “messengers.” BXD has certain regulatory effects on neurotransmitters and can provide a more effective treatment for some CNS diseases.

Depression can reduce gastrointestinal motility, change the regulatory functions of the related gastrointestinal nerves, and promote the development of ulcers, even intestinal cancer. Additionally, these gastrointestinal complexions will result in a vicious cycle by aggravating the anxiety and depression of patients in turn. By using the biological module analysis of network pharmacology, Yu et al predicted the active ingredients, key targets, and signaling pathways of BXD that are engaged in the treatment of UC and depression. Multiple biological functions including lipid metabolism, inflammatory response, oxidative stress response, insulin secretion regulation and estradiol response mainly via NF-κB signaling pathway, HIF-1 signaling pathway, the regulation of 5-hydroxytryptamine synaptic, and arachidonic acid metabolism were found.^[57]^

In the early stages of Alzheimer’s disease, deficient energy metabolism and less glucose utilization has been occurring and associated with impaired cognition, which suggested that Alzheimer’s disease is a metabolic disease related to brain insulin/insulin-like growth factor resistance. A study investigated the effects of BXD on cognitive deficits in APPswe/PS1dE9 double transgenic mice and suggested a potential mechanism, which included increasing the quantity and ultrastructure of synaptic to improve cognition, reinforcing insulin signaling and elevating the expression of glucose transporter 1 and glucose transporter 3 levels.^[82]^

Recent studies have shown that some digestive diseases, such as gastric ulcer and GERD, are closely related to endogenous brain-gut peptides. Recently, by comparing the variations in the content of both gastric mucin and hypothalamic monoamine, the effect of BXD on water-immersion restraint stress-induced gastric ulcer rats were well demonstrated.^[92]^ Somatostatin, as a typical brain-gut peptide widely distributed in brain, pancreas, intestinal nerve cells and gastric mucosal endocrine D cells, plays a vital role in stabilizing the internal environment. According to another study, BXD can increase the expression of somatostatin to realize its therapeutic efficacy on stress gastric ulcer.^[91]^ Additionally, the pathogenesis of gastric ulcer has been reported to be associated with the decrease of concentration and mRNA expression level of Leptin, and the increase of concentration and mRNA expression level of Endothelin-1.^[109]^ It was found that BXD could upregulate Leptin and inhibit Endothelin-1 to accelerate the healing of gastric ulcer.^[90]^ Moreover, by analyzing the correlation between the degree of esophageal mucosal injury and the content of neurotensin in rats, researchers found neurotensin may be a key factor in the progress of GERD. And one of the possible mechanisms of BXD in treating GERD might involve the regulation the synthesis and secretion of neurotensin.^[86]^

On the other hand, some patients suffering digestive diseases may have conditions regarded as non-ulcer dyspepsia. They are frequently subjected to affective stress, which can result in anomalies in the HPA axis and autonomic nerve function. Additionally, the release of neuropeptide Y can also induce the abnormalities of HPA axis activity.^[110]^ To explore the effects of BXD on the HPA axis and autonomic nervous function, researchers placed volunteers under artificial stress by venipuncture. The results showed that BXD could suppress the levels of neuropeptide Y, plasma adrenocorticotropic hormone, and cortisol under stress, which may be helpful for treating diseases induced by stress.^[97,98]^

## 14. Anti-bacterial

The infection or imbalance of bacterial in vivo can activate inflammatory responses or immune systems, which contributes to the pathological progress of various systems including CNS, gastrointestinal tract, and endocrine.

Hp is a common pathogen of gastrointestinal diseases (such as gastritis, gastric ulcer, duodenal ulcer, even cancer). Recent studies have found that BXD has certain effects on gastrointestinal diseases caused by Hp infection.^[14]^ In Hp-treated GES-1 cells, Chen et al found that treatment with the BXD-containing serum could suppress the expression of TGF-β1 and Smad3, and increase the expression of Smad7 in a dose-dependent manner.^[70]^ Qu et al tested the antibacterial effect of BXD on the 8 strains of Hp resistant strains separated from patients. The results demonstrated that BXD had strong bacteriostatic effect on the bacteria resistance of Hp strains in vitro, suggesting that it may be employed as a therapeutic choice for resistant Hp-related gastritis.^[71]^

## 15. Gut microbiota-modulating

The rise in pathogenic bacteria in the intestines has been associated with an intestinal ecology imbalance in patients with constipation. A study examined the therapeutic effect of BXD on mice with spleen-deficiency constipation showed that the intestinal micro-ecological balance could be regulated by BXD via promoting the growth of *bifidobacterium* and restoring the amount of *colibacillus* and *lactobacillus*. By analyzing enzyme activities in mice intestine, researchers found that BXD significantly decreased the activity of protease, increased the activity of xylanase and amylase, suggesting that BXD could improve the digestive function of mice while increase the activities of the enzymes secreted by intestinal microbes. Overall, BXD had therapeutic effect on spleen-deficiency constipation by balancing the activity of some enzymes and intestinal bacteria.^[99]^ Moreover, Zhao et al explored the possible mechanism of modified BXD in the treatment of polycystic ovarian syndrome with insulin resistance (PCOS-IR). By analyzing the characteristics of the intestinal microbiota, they found that genus *Clostridium_sensu_stricto_1* might exert a pivotal part in PCOS-IR as pathogenic bacteria. Modified BXD may alleviate PCOS-IR via balancing intestinal microbiota and enhancing metabolic disorders.^[100]^

## 16. Clinical applications

BXD has shown promising pharmacological and clinical effects in the treatment of multiple gastrointestinal diseases such as tumors, gastritis and Hp infection. Adjusting the dosage and drug compositions to accommodate inter-individual variability in diseases and physique to demonstrate stronger clinical significance and further expand its clinical applications.

## 17. Application of BXD as a complementary in treatment of malignant tumor

Currently, the toxicity and adverse effects of radiotherapy and chemotherapy in the progress of treating malignant tumor are crucial factors that cause anorexia, emaciation, weakness, and even shortened survival times. As the 3 primary effects of bone marrow suppression, leukocytopenia, thrombocytopenia, and erythrocytopenia could lead to weakness, exhaustion, and loss of immunity, influencing the quality of life and prognosis of cancer. A study showed that BXD combined with afatinib treatment can help delay the progression of gallbladder cancer.^[80]^ A systematic review used meta-analysis to evaluate the effectiveness and safety of BXD adjuvant therapy for GC indicated BXD, whether used alone or combined with radiotherapy and chemotherapy, can improve in the treatment of GC.^[51]^ Additionally, in a prospective non-randomized control study, BXD combined with chemotherapy was found to significantly relieve clinical symptoms, reduce chemotherapy associated adverse effects, improve quality of life (QoL), and prolong disease-free survival of patients with stage III colon cancer (cold-heat complicated pattern).^[95]^

The most prevalent and fatal kind of liver cancer is hepatocellular carcinoma (HCC). Owing to the scarcity of efficient treatments and the high rates of recurrence and metastasis, HCC often ends with an awfully poor prognosis. Presently, BXD is frequently used as a complementary or alternative treatment for liver cancer, to lessen the side effects of conventional therapies, to relieve symptoms, and to enhance quality of life. In a clinical study, 68 patients with advanced HCC received BXD treatment for at least 1 month. The results are promising since no drug-related serious adverse events were observed during the study and can be useful for treating patients with advanced HCC in a practical therapeutic environment.^[78]^

## 18. Application of BXD in treatment of adverse reactions of anticancer drugs

For extensive-stage small cell lung carcinoma (SCLC), CPT-11 can be utilized as a first-line therapeutic medication in both first-line and second-line therapy.^[111]^ Unfortunately, the delayed diarrhea caused by CPT-11 limits its clinical application. BXD was beneficial in alleviating and preventing CPT-11-induced diarrhea, according to a randomized comparative trial analysis of its curative efficacy when paired with chemotherapy for advanced NSCLC.^[112]^ A total of 27 patients with recurrent SCLC who were receiving chemotherapy regimens including CPT-11 were enrolled in another study. After receiving BXD orally for 5 consecutive days before the second cycle of chemotherapy, 4 of 5 patients with delayed diarrhea had relief or were under control.^[3]^ Furthermore, by utilizing UPLC to analyze 7-ethyl-10-hydroxycamptothecin (SN-38) levels in mice treated with a variety of BXD combinations, Shi et al found the relationships of BXD spectrum-effect and identified the components in BXD (including chrysin, coptisine, wogonoside, baicalin, berberine, palmatine and chrysin-7-O-glucuronide) that were bioactive in preventing CPT-11-induced diarrhea in mice.^[73]^ As a side effect of BXD, constipation occurred in 10% of patients in the study by Sakata et al^[113]^ and in 11% of patients in the study by Mori et al,^[112]^ although they were all mild grade 1.

## 19. Application of BXD in treatment of GERD

A systematic review of controlled trials, including randomized controlled trials (RCTs) of BXD as a treatment for GERD, in eleven databases, revealed the effectiveness and safety of BXD for the treatment of GERD.^[83]^ Another meta-analysis based on previous systematic reviews/meta-analyses further provided evidence that BXD had promising efficacy to treat GERD patients.^[88]^ In treatment of 40 cases of laryngopahryngitis caused by GERD, it has been found in a parallel study that the therapeutic effect of modified BXD is significantly more effective than Jinsang Liyan Wan (Pills for a good voice by relieving sore-throat).^[87]^ However, Dai et al did find side effects during the treatment, such as diarrhea, abdominal distention, nausea, etc. However, these side effects had negligible impact on the experiment.^[84]^

## 20. Application of BXD in treatment of FD

According to a study comparing BXD to a placebo in patients with FD, though BXD showed no significant improvement in the symptom-related Nepean dyspepsia index score and the QoL related scores in Nepean dyspepsia index and FD-QoL after 4 weeks of treatment, it did significantly improve the visual analog scale score and fullness after eating-related symptoms in the follow-up visit. No serious adverse events but 8 cases of mild adverse events were reported during the trial.^[7]^ BXD has also been used to treat postprandial distress syndrome, a subgroup of FD, is known as the Wei-Pi syndrome in TCM traditionally. In a clinical trial, 84 patients with Wei-Pi syndrome were randomized 1:1 into the BXD or waitlist control group. The outcome demonstrated the effectiveness of modified BXD in enhancing the clinical symptoms and QoL of Wei-Pi syndrome patients.^[65]^

## 21. Application of BXD in treatment of gastritis and gastric precancerous lesion

Gastric precancerous lesions including CAG with dysplasia and/ or intestinal metaplasia. Recently, using BXD to treat gastric precancerous lesions has been the subject of many RCTs. Yi et al performed a thorough search of the literature on the use of BXD in the treatment of gastric precancerous lesions and summarized the overall effectiveness and safety of BXD thru qualitative or quantitative analysis. The findings provided relevant clinical guidelines for gastric precancerous lesions.^[63]^ Likewise, for CAG patients, another meta-analysis revealed that compared to the control group, BXD was safer and more efficient.^[60]^ Moreover, Xia et al has been adjusting the dosages of BXD herbal pairs dynamically according to the concrete condition and treated 98 cases of CAG. Results showed better therapeutic effects in treatment group than those in the control group.^[64]^

## 22. Application of BXD in treatment of Hp-associated gastrointestinal diseases

Helpful evidence regarding the effectiveness and safety of BXD in the treatment of Hp-associated gastrointestinal diseases has been provided by a systematic review. It was indicated that BXD is useful in the treatment of Hp positive peptic ulcers by concentrating on the comprehensive and systematic summary of the existing clinical evidence.^[69]^ Another study compared the effectiveness and safety of BXD as alternative therapy versus standard triple therapy or quadruple therapy for patients with peptic ulcer or chronic gastritis infected with Hp. Based on the findings, the cure rate and effectiveness rate were slightly better in the BXD alone group than in the conventional therapy group for patients with peptic ulcer or chronic gastritis infected with Hp.^[72]^ Adverse events appeared in 3 participants in the BXD group and 26 participants in the conventional therapy group.

## 23. Application of BXD in treatment of other diseases

To assess the current clinical evidence of BXD for DGP, researchers included RCTs using BXD/modified BXD for DGP.^[77]^ The results showed that the effect of BXD for DGP was more effective than the control group in 16 RCTs involving 1302 patients. And only one trial recorded adverse events, no obvious adverse event occurred, which demonstrated the effectiveness and safety of BXD for the treatment of DGP. As an idiopathic and nonspecific inflammation of the ileal pouch, pouchitis is the most common complication after restorative proctocolectomy with ileal pouch-anal anastomosis (a standard operation for patients with UC). Given that diarrhea is the primary symptom of pouchitis, BXD may be an available treatment for patients with chronic pouchitis. In a pilot research, the effectiveness and safety of BXD in treating chronic pouchitis were assessed.^[58]^ By enrolling 19 patients with chronic pouchitis and treating them with BXD, the pouchitis DAI of 8 patients was reduced to below 7. As to the mean total DAI scores, they considerably dropped from 11 ± 2.5 to 6.5 ± 2.5 (*P* < .001). The mean total dose of the antimicrobial medication ciprofloxacin reduced noticeably from 491.6 ± 182.4 mg/kg to 392.5 ± 184.0 mg/kg (*P* < .05). With no severe adverse events noted, the outcome indicated that BXD is safe and effective to treat chronic pouchitis.

## 24. Adverse effects

Generally, BXD is widely recognized as safe and effective. Up to date, there has not been systematic study focusing on its toxicity. However, the methodological quality of included studies is low, and long-term efficacy and safety are still uncertain, which indicates that the findings above should be read with caution. Although the prevalence has not been quantified, side effects of BXD included liver function failure, pseudo hyperaldosteronism and interstitial pneumonia. Thereby, for clinical application, BXD should not be administered aimlessly in the long term (and follow-up laboratory tests and radiography should be performed as appropriate). Well-designed, large-scale, and high-quality randomized controlled clinical trials with scientific rigor are warranted for stronger evidence in future research.

## 25. PK investigation

Pharmacokinetics (PK) is a discipline which investigates quantitatively the law of in vivo absorption, distribution, metabolism as well as excretion of medicine, and explains the law of blood drug concentration with time by utilizing mathematical principles and methods. Obviously, it is the key approach to uncover the obscure pharmacodynamic characteristics and toxicity of TCM herbs or formulae.

As a traditional Chinese prescription, BXD has different kinds of PK interactions among its multi-components. Recently, studies of the PK profiles and absorption of alkaloids, flavonoids and saponins in both pure components and BXD have been well performed.^[5,114]^ Wang et al developed a rapid and highly sensitive LC-MS/MS approach to simultaneously determinate 9 active ingredients (baicalin, baicalein, wogonoside, wogonin, scutellarin, berberine, coptisine, ginsenoside Rb_1_ and ginsenoside Re) in rat plasma after BXD administration orally. In line with previous studies,^[115–117]^ all of the 5 flavonoids showed the double-peak phenomenon. The peak concentration (C_max_) and area under the concentration-time curve (AUC_(0–t)_) values of baicalin, baicalein, wogonoside, wogonin and scutellarin after oral administration of BXD were significantly higher, compared with those of the HQ extract or scutellarine compound,^[118]^ indicating better absorption after administration with BXD. The phenomena might be due to mutual interactions of multiple constituents of BXD like ginsenosides, licorice flavonoids and saponins.

Additionally, after oral administration of BXD to rats, multiple absorption peaks were observed in the plasma concentration-time curves of berberine and coptisine, which may be the result of site-specific absorption rather than enterohepatic circulation. The absorption of the alkaloids was significantly enhanced in herbal prescriptions compared to the earlier pharmacokinetic investigations.^[119,120]^ Some constituents in BXD such as baicalin may act as a P-glycoprotein-inhibitor to promote the intestinal absorption of HL alkaloids.^[121]^ The inhibition of cytochrome P450 enzymes by compounds in BXD may lead to a decrease in the rate of metabolism and an increase in the absorption of berberine and coptisine in vivo.^[115]^

As to saponins, ginsenoside Rb_1_ only exhibited one single peak (at around 6 h) in the mean plasma concentration-time profile, which was in line with previous reports that revealed the low bioavailability of ginsenosides after administration orally.^[122,123]^ According to current study, dose-modified C_max_ and AUC_(0-t)_ values of ginsenoside Rb_1_ showed an enhancement in absorption by co-administrating with other crude herbs of BXD. Instead of the reduced clearance in vivo, these phenomena might be attributed to the weakened metabolism of ginsenoside Rb_1_.^[124,125]^ Further research on comparative pharmacokinetics of active components of ZGC, including liquiritin, liquiritigenin, isoliquiritin, isoliquiritigenin, glycyrrhizic acid and glycyrrhetinic acid, in rats after BXD and its various compatibilities administration orally was also conducted.^[114]^ The findings show that several PK characteristics of the active compounds were impacted by the compatibility of other herbs of BXD, and the bioavailability of most ingredients are increased in whole formula.

There were few studies on the PK investigation of BXD under disease states. Recent research by Zhou et al revealed systematic PK data of BXD under the pathological settings of chronic gastritis.^[126]^ In comparison to the control group, the PK parameters of baicalin, wogonoside, liquiritin, and liquiritigenin in rats with chronic gastritis altered dramatically, with significant reductions in C_max_, AUC_(0-t)_, and AUC_(0-∞)_. The T_1/2_ of wogonoside and liquiritin were also considerably decreased in rats with chronic gastritis. These findings could offer some useful references for BXD in the clinical treatment of gastritis.

Relatively, common analytical techniques used in PK investigations typically need large amounts of sample. To explore the PK of baicalin in BXD and various compatibilities in mice, an indirect competitive enzyme-linked immunosorbent test based on monoclonal antibodies against baicalin was established and effectively employed.^[127]^ A technique with improved detection sensitivity would thus be very beneficial for PK research, particularly in small animals.

## 26. Conclusion

**Figure 3.**
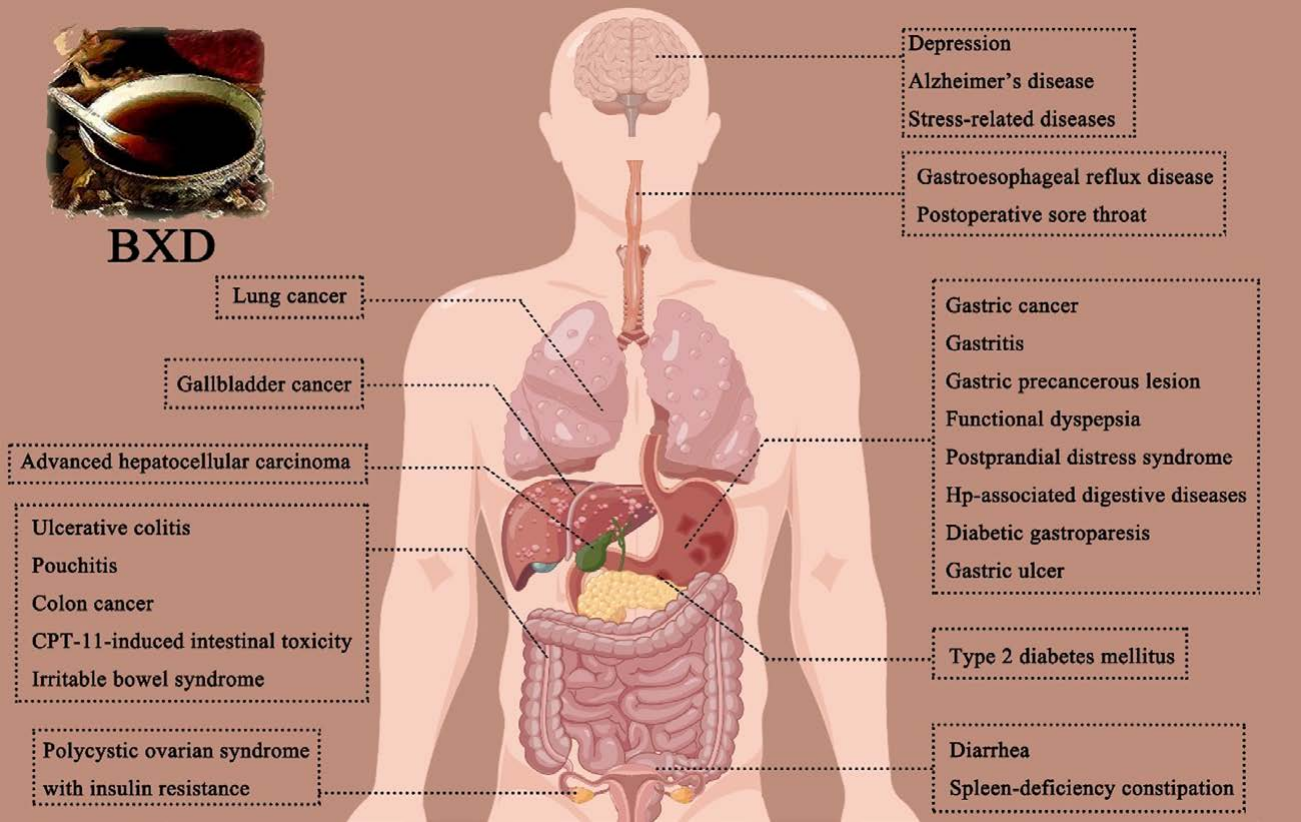
Schematic diagram of diseases that BXD is effective against. BXD = Banxia Xiexin decoction.

Here, we review the phytochemical, pharmacological, clinical, and PK investigations of BXD. The potential active ingredients in BXD can be classified as alkaloids, flavonoids and saponins. Among them, berberine, baicalin, and ginsenoside Rb_1_ are the representative constituents. With abundant constituents, BXD shows pharmacological activities in multiple parts, including anti-tumor, gastrointestinal motility, anti-inflammatory, blood glucose and insulin resistance modulating, CNS modulating, anti-bacterial, and gut microbiota-modulating. Clinical investigations and systematic reviews have shown that BXD is helpful against malignant tumors, side effects of anticancer medications, GERD, FD, gastritis, and gastric precancerous lesions, as well as chronic pouchitis and gastrointestinal conditions associated with Hp (Fig. 3). Some significant PK parameters reflect the key variations between the PK profiles of the main constituents in BXD and pure drugs. BXD tends to exhibit greater pharmacological effects than single- or couplet-drug therapies. These findings show the potential interaction between the co-occurring components in BXD.

To better illuminate the compositive principle and action features of BXD, challenges remain. These challenges also, to a certain degree, represent the common problems of TCM. First, precisely comprehending the classical literatures on the application of BXD together with completely randomized blank controlled double-blind clinical trials will aid in confirming the clinical effects and exposing the negative reactions. Second, customized medicine is a distinctive feature of TCM, according to which one single formula may be used to treat various diseases with comparable. Then, studies of clinical syndromes and the subsequent development of preclinical research systems in cell or animal with reliable pathological features or biomarkers are anticipated to reveal the rationality and principle of compatibility of monarchs, ministers, assistants, and ambassadors in TCM prescription, to unveil the ideas of TCM theories such as acrid-opening, bitter down-bearing and sweet-tonification. Furthermore, it is difficult to identify the exact active ingredients and the pathophysiological process node that has been interfered with. TCM formula frequently rely on abundant constituents to exert their therapeutic effects by activating or inhibiting a variety of targets. To investigate the network of interactions between the various components and the multiple targets, high-throughput screening on the targets related to the representative signaling pathway and further pharmacological assays on the synergistic impact of those compounds are necessary. Based on these digital database resources, it is also necessary to study the interactions between the chemicals and targets and relationship between the targets using system pharmacology, which facilitates the prediction of the potential activate ingredients and the underlying targets or signaling pathways of TCM formula. Rigorous biochemics and pharmacologics, proteomics, transcriptomics, and metabolomics are needed to validate literature mining results.

## Author contributions

**Writing – original draft:** Zehua Zhou.

**Writing – review & editing:** Rui An, Lisha You, Kun Liang, Xinhong Wang.
